# Assessing the patient’s affective perception of their psychotherapist: validation of the *in-Session Patient Affective Reactions Questionnaire*

**DOI:** 10.3389/fpsyt.2024.1346760

**Published:** 2024-04-24

**Authors:** Alberto Stefana, Paolo Fusar-Poli, Eduard Vieta, Eric A. Youngstrom

**Affiliations:** ^1^Department of Brain and Behavioral Sciences, University of Pavia, Pavia, Italy; ^2^Department of Psychology and Neuroscience, University of North Carolina at Chapel Hill, Chapel Hill, NC, United States; ^3^OASIS Service, South London and Maudsley NHS Foundation Trust, London, United Kingdom; ^4^Early Psychosis: Interventions and Clinical-detection (EPIC) Lab, Department of Psychosis Studies, Institute of Psychiatry, Psychology and Neuroscience, King’s College London, London, United Kingdom; ^5^Bipolar and Depressive Disorders Unit, Hospital Clinic, Institute of Biomedical Research Agusti Pi i Sunyer (IDIBAPS), Centro de Investigacio Biomédica en Red de Salud Mental (CIBERSAM), University of Barcelona, Barcelona, Spain; ^6^Institute for Mental and Behavioral Health Research, Nationwide Children’s Hospital, Division of Child and Family Psychiatry, The Ohio State University, Columbus, OH, United States; ^7^Helping Give Away Psychological Science, Chapel Hill, NC, United States

**Keywords:** therapeutic relationship, affective reaction, emotional reaction, in-session process, self-report measure, psychotherapy, psychological assessment, evidence-based assessment

## Abstract

**Background:**

Psychotherapists need effective tools to monitor changes in the patient’s affective perception of the therapist and the therapeutic relationship during sessions to tailor therapeutic interventions and improve treatment outcomes. This study aims to evaluate the factor structure, reliability, and validity of the *in-Session Patient Affective Reactions Questionnaire* (SPARQ), a concise self-report measure designed for practical application in real-world psychotherapy settings.

**Methods:**

Validation data was gathered from (*N* = 700) adult patients in individual psychotherapy. These patients completed the SPARQ in conjunction with additional measures capturing sociodemographic details, characteristics of therapeutic interventions, individual personality traits, mental health symptom severity, elements of the therapeutic relationship, and session outcomes. This comprehensive approach was employed to assess the construct and criterion-related validity of the SPARQ.

**Results:**

The SPARQ has a two-factor structure: Positive Affect (*k* = 4, *ω* total = .87) and Negative Affect (*k* = 4, *ω* total = .75). Bifactor confirmatory factor analysis (CFA) yielded the following fit indices: *X*^2^[*df*] = 2.53, CFI = .99; TLI = .98; RMSEA = .05; and SRMR = .02. Multi-group CFAs demonstrated measurement invariance (i) across patients who attended psychotherapy sessions in person *versus* in remote mode, and (ii) across patients with and without psychiatric diagnoses confirmed metric invariance. Furthermore, the SPARQ showed meaningful correlations with concurrently administered measures.

**Discussion:**

The SPARQ proves to be a valuable instrument in clinical, training, and research contexts, adept at capturing patients’ session-level affective responses towards their therapist and perceptions of the therapeutic alliance. Comprehensive descriptive statistics and a range of score precision indices have been reported, intended to serve as benchmarks for future research.

## 1 Introduction

Emotions are a fundamental component of the human experience. They are the result of an evolutionary process aimed at helping people mobilize their organism to deal with important interpersonal encounters and, more generally, with fundamental life tasks (1, 2). Therefore, it is not surprising that emotions have been considered therapeutically significant since the origins of psychotherapy (3), whether they were part of the clinical manifestation of the patient or part of the therapist’s experience in the therapeutic effort to help them (4).

### 1.1 A brief historical background

Historically, the phenomenon of the emotions experienced by a patient towards their clinician was first theoretically conceptualized by Sigmund Freud, who introduced the term transference towards the end of the 19^th^ century (5). Freud described transference as the redirection of a patient’s feelings, fantasies, desires, and even entire scenarios, which are re-enactments of past psychological experiences with significant figures from childhood, onto the clinician. He identified two coexisting forms of transference: positive transference, which involves conscious affectionate feelings, and negative transference, which encompasses hostile feelings that usually remain unconscious.

Building on Freud’s work, psychoanalyst Melanie Klein and her colleagues expanded the definition of transference in the second half of the 20^th^ century (6). They viewed it as both a conscious and unconscious manifestation of past and present experiences, relationships, emotions, thoughts, and fantasies, encompassing both positive and negative aspects in relation to the clinician. This broader interpretation was termed the ‘total situation,’ and aimed to include all facets of the patient’s relationship with the clinician in the concept of transference (7).

In contemporary understanding, transference is generally recognized by clinicians and researchers as a pattern of enduring emotions, thoughts, motivations, and behaviors that are activated and displayed in the patient’s relationship with the therapist (8, 9). Based on this conceptualization of the construct, we developed the *in-Session Patient Affective Reactions Questionnaire* (SPARQ) (10) to measure in a self-report format patterns of affective, cognitive, and behavioral responses experienced by a patient toward their therapist during an individual psychotherapy session.

### 1.2 Pantheoretical nature of the in-session emotions

Many clinicians believe that one of the main differences between analytic and nonanalytic psychotherapies lies in the theoretical and clinical attention the former place upon transference. This is one of the most common and persistent misconceptions, particularly about CBT (11–13). In fact, Aaron Beck (14), discussing similarities and differences between cognitive therapy and psychoanalysis, maintained that the former ‘has access to the types of ideational material obtained in free association, dream reporting, and in the patient’s reactions to the therapist (transference). However, by staying close to the data, the therapist avoids becoming enmeshed in the abstract speculation of psychoanalysis’.

It is worth noting that leading theoreticians of nearly all of the major psychotherapeutic approaches acknowledge that the phenomenon psychoanalysts call transference exists in their therapies, although they might name it differently and vary markedly in how clinically important they consider it, as well as in if and how it should be dealt with (15). Transference has been documented by such authors as Carl Rogers (16), within the client-centered therapy, Fritz Perls (17), within gestalt therapy, and Rollo May (18) within existential therapy. In the CBT model, Beck (14, 19) talked explicitly of transference, Goldfried and Davison (20) described it in terms of ‘parataxic distortion.’ Other CBT therapists define it as overgeneralization (21). Francine Shapiro (22) underlines that the decision to proceed with EMDR in case of a patient’s dissociative disorder depends, among other factors, on the therapist’s ability to anticipate and accommodate transferences.

### 1.3 Emotional expression in psychotherapy

Research on emotional expression in psychotherapy provides evidence that emotions substantially contribute to therapy outcomes at both the session and treatment levels (23–25). Furthermore, emotions are an element of both therapy-specific and nonspecific therapeutic processes related to clinical efficacy (26, 27). Helping patients become aware of their emotions and make constructive use of them appears to be important in therapeutic change (28, 29). To achieve this therapeutic result, the therapist has to collaboratively focus on the emotions experienced by the patient during the session, helping them to recognize, feel, tolerate, explore, accept, regulate, make sense of, transform, and manage these emotions (30).

Therapy can reactivate repressed or denied emotions, allowing the patient to recover undesired experience, and activate new emotions. Both situations give therapy participants information about the patient’s needs and responses to specific intrapsychic and interpersonal situations, and thus represent an opportunity to work through previously denied and dreaded feelings and incorporate new experiences into those generated in the past (31). This helps patients transform their persistent memory-based problematic implicit emotional procedures and, in turn, change their patterns of interactions with the environment (32). Among all the emotions experienced by a patient in the ‘here and now’ of the therapeutic session, those felt toward the therapist are particularly important and useful for fulfilling or facilitating the therapeutic work just described (33, 34). An empathic and affirming therapeutic relationship is a pivotal factor in whether emotional experience and expression are productive *versus* negative for therapy (24). It is both a key ingredient in change and a prerequisite for the effective implementation of psychotherapeutic work (35).

Therefore, therapists need to monitor (assess) even small changes in the patient’s affective perception of the therapeutic relationship as they occur to address and repair the relationship. Without assessing the perception of the patient, one cannot intervene promptly and appropriately. Although various measures have been developed to assess emotions and emotional expression – both as state and trait constructs [DES (36), PANAS (37), POMS (38, 39)] – and several have been used in the context of psychotherapy research (24, 40), only a few of them formally incorporate attention to affective processes in dyadic therapy relationships. Currently, the few existing psychological assessment tools that measure patients’ emotional reactions towards their therapists are primarily therapist-rated. These tools assess transference in the narrow sense, as defined by Freud (e.g., 41, 42), or in the broader sense of the ‘total situation’ (e.g., 43). However, studies investigating this phenomenon from the patient’s perspective have utilized scales not specifically designed for examining response patterns within the psychotherapeutic setting or are length (e.g., 44). For example, the Psychotherapy Relationship Questionnaire (43) is a clinician-report scale that measures the same construct as SPARQ, but it is composed of 90 items and assesses affective reactions over a time frame of approximately eight sessions. Existing self-report measures that capture constructs such as alliance (45, 46) or alliance rupture (47) only indirectly include affective content.

To fill this gap, we wrote a large item set focusing on affective responses during the psychotherapy session. The initial pool contained more than 130 items. We revised based on expert review, and then used a sequence of exploratory and confirmatory factor analyses to understand the dimensionality. Item response theory analyses then selected the items providing the most information for each factor, resulting in the two four-item subscales that we are evaluating in terms of construct validity in the present study (10).

### 1.4 Aim

The purpose of the current study is to validate a brief self-report measure of the patterns of thought, feeling, and behavior experienced in session by the patient toward their therapist, which is clinically sophisticated, pantheoretical, and feasible to be administered in a real-world psychotherapy setting.

Specific aims included selecting items that balanced brevity, in order to improve tolerability and reduce missing data or reactance (48, 49), while also looking at item characteristics to improve information value when dyads start to experience affective reactions. We also aimed to develop balanced scales for positive and negative valenced affect, and we calculated person-centered benchmarks for clinically significant changes in scores (50, 51). Provided that the other evidence of construct validity appears sufficient, these patient-centered benchmarks provide a good foundation for clinical use. We extended our prior work by also testing a bifactor model to see if it provided better fit, indicating that the subscales might represent distinct but related constructs [as has been shown with the subscales of the RRI (52)]. Because our data collection happened after the COVID pandemic (53), we had an opportunity to compare whether the scales showed comparable psychometrics when used for in-person *versus* online therapy. We tested this ancillary aim through progressive structural invariance model comparisons (54).

We examined several aspects of construct validity by looking at correlations with demographic feature such as age, sex, ethnicity, and education (all expected to have small, often nonsignificant correlations with session-level affective response, e.g., |*r|* ~ .2 or smaller), as well as clinical diagnoses that were the focus of treatment, and session parameters. These were expected to also be small, in as much as the items were designed to be session-focused.

Construct validity was further explored by examining correlations with the ‘big five’ personality traits (where we anticipated moderate positive correlations between positive affect and extraversion and agreeableness, and moderate positive correlation between trait negative emotionality/neuroticism and session-level tendencies towards negative affect) (55), and moderate correlations with self-functioning and interpersonal functioning. We also included state measures of affect, as well as widely used scales of depressive and anxious symptoms. A large body of work on the tripartite model of depression and anxiety indicates that these types of scales share a common dimension of negative, whereas depression also includes low positive affect features such as anhedonia and loss of interest (although depression scales frequently under-represent the low-PA content, increasing the apparently overlap with NA and thus with anxiety). Because these also are state scales, and because they are dimensional instead of discrete yes/no items, we hypothesized that correlations would be larger than observed with diagnoses, but still moderate sized (*r* values in the.3 range).

Finally, we also examined convergent validity with measures of therapeutic relationship, including working alliance, real relationship genuineness and realism, and session outcome ratings. Here we expected large correlations (*r* > .5, but ideally < .85, or else more than 70% of the variance would be shared, raising the question of whether the measures were redundant) (56, 57).

## 2 Methods

### 2.1 Recruitment

The target population was adult patients fluent in English with heterogeneous mental conditions and in different types and timings of psychotherapeutic treatment. Participants were recruited via two online patient registers, i.e., Research for Me ResearchMatch, from March through April 2023. After providing informed consent, they completed a one-time battery of measures implemented on Qualtrics software. This research was approved by the Institutional Review Board of the University of North Carolina at Chapel Hill (Study #: 23-0216; Approval Date: 3/06/2023). Online informed consent was provided by all participants.

### 2.2 Participants

The sample was composed of 700 adult psychotherapy patients. Most (81%, *n* = 564) were female. The most common age range was 30 to 39 years (28%, *n* = 193), followed by 23 to 29 years (20%, *n* = 142). Most participants had a psychiatric diagnosis (84%, *n* = 590). The most prevalent DSM diagnosis among the participants was anxiety disorder (66%, *n* = 464), followed by unipolar depressive disorder (56%, *n* = 391) and trauma- and stressor-related disorders (35%, *n* = 244). Approximately half of the subjects had three or more diagnoses at the diagnostic category level (51%, *n* = 302), one-third had two diagnoses (31%, *n* = 185), and the remainder had a single diagnosis (18%, *n* = 103). A significant portion of patients had been in psychotherapy for over 24 months (47%, *n* = 332), with most attending two to four sessions per month (77%, *n* = 543). Over half of the participants (53%, *n* = 369) conducted their most recent session via video call. **Table 1** reports sample sociodemographic, clinical, and treatment characteristics. All information, including psychiatric diagnosis, was self-reported.

**Table 1:** Demographics, clinical, and treatment characteristics of participating patients (*N* = 700).

Demographics	% (*n*)
Age (years)	
18–22	9% (66)
23–29	20% (142)
30–39	28% (193)
40–49	16% (109)
50–59	14% (99)
≥60	13% (91)
Biological sex	
Female	81% (564)
Male	18% (128)
Intersex	0% (1)
I prefer not to say	1% (7)
Gender	
Woman	74% (512)
Man	19% (132)
Other	4% (29)
Woman–Other	2% (14)
Man–Other	0% (3)
I prefer not to say	1% (6)
Education	
Less than high school	0% (2)
High school graduate	3% (24)
Some college	19% (136)
2-year degree	9% (64)
4-year degree	33% (231)
Professional degree	28% (195)
Doctorate	7% (48)
Ethnicity	
White	81% (566)
Black or African American	10% (68)
Asian	4% (29)
Native Hawaiian or Pacific Islander	1% (4)
American Indian or Alaska Native	1% (5)
Other	4% (28)
Clinical characteristics ^a^	
Diagnoses	
Any psychiatric disorder	84% (590)
Any anxiety disorder	66% (464)
Any (unipolar) depressive disorder	56% (391)
Any trauma- and stressor-related disorders	35% (244)
Any neurodevelopmental disorder	24% (165)
Any bipolar or related disorder	13% (88)
Any eating disorder	10% (71)
Any other psychiatric disorder	6% (45)
Any disruptive behavior and dissocial disorder	2% (15)
Schizophrenia or any other psychotic disorders	1% (9)
Any cluster A personality disorder	0% (3)
Any cluster B personality disorder	6% (43)
Any cluster C personality disorder	6% (41)
Treatment characteristics	
In psychotherapy from	
0 to 3 months	14% (99)
4 to 6 months	14% (96)
7 to 12 months	11% (79)
13 to 24 months	13% (94)
>24 months	47% (332)
Session frequency	
1 or less per month	19% (130)
2 to 3 per month	39% (276)
1 per week	38% (267)
2 or more per week	4% (27)
Session attendance	
Video call	53% (369)
In person face to face	36% (251)
Telephone call	8% (59)
In person on the couch	3% (21)
Therapy location	
Private practice	70% (493)
Private health institution	11% (76)
Public health institution	10% (67)
University counseling center	4% (26)
Other	5% (38)
Therapist biological sex (Female)	81% (565)

a *N* sums to more than 700 because cases could have more than one diagnosis.

### 2.3 Measures

A battery designed to evaluate convergent and discriminant validity included measures of patient characteristics, personality traits, mental health status, aspects of the therapeutic relationship, and session outcome. The ‘sociodemographic, clinical, and treatment domain’ collects information about the sociodemographic and clinical characteristics of the patient, as well as the psychotherapeutic treatment they receive. The ‘trait domain’ focuses on individual personality traits. The ‘mental health state domain’ encompasses measures of symptoms that impact the daily lives of participants. These three domains are used to evaluate discriminant validity. The ‘therapeutic relationship domain’ provides insights into the nuances of the patient-therapist relationship from the patient’s perspective, crucial for assessing the convergent validity. Finally, the measure of the ‘session outcome domain’ is instrumental in determining predictive validity. We prioritized previously validated short forms where available to reduce respondent burden, increasing participation rates and data completeness, in keeping with best practices (58).

#### 2.3.1 Sociodemographic, clinical, and treatment domain

The participants completed an 11-item sociodemographic and clinical data form, which recorded the information listed in **Table 1**.

#### 2.3.2 Trait domain

##### 2.3.2.1 Big Five Inventory–2-Extra-Short form

The BFI-2-XS (59) is a 15-item self-administrated scale used to assess personality at the level of the Big Five domains (three items for each domain). Items are rated on a 5-point Likert scale ranging from 1 = ‘Disagree strongly’ to 5 = ‘Agree strongly.’ In our internal consistency analysis, average inter-item correlations were .34 for the Extraversion dimension, .27 for the Agreeableness dimension, .38 for the Conscientiousness dimension, .35 for the Negative Emotionality dimension, and .25 for the Open-Mindedness dimension. The BFI-2-XS has been used extensively, with more than 800 citations in Google Scholar at the time of preparing this paper.

##### 2.3.2.2 The Level of Personality Functioning Scale-Brief Form 2.0

The LPFS-BF 2.0 (60) is a 12-item self-report questionnaire for the assessment of the severity of personality pathology. It assesses impairment in self-functioning and interpersonal functioning based on levels of personality functioning described in Section III of DSM-5. Ratings are made on a 5-point Likert scale, ranging from 1 = ‘Completely untrue’ to 4 = ‘Completely true.’ Higher scores indicate greater impairment. The Cronbach’s alpha coefficient for our sample was.85.

#### 2.3.3 Mental health state domain

##### 2.3.3.1 International Positive and Negative Affect Schedule – Short Form

The I-PANAS-SF (61) is a 10-item self-report measure of the frequency with which the respondent has experienced positive (5 items) and negative (5 items) affects during the last week. The ten emotional adjectives are rated on a five-point Likert scale ranging from 1 = ‘Very slightly or not at all’ to 5 = ‘extremely’). Higher scores indicate strong emotional activation. Reliability coefficients in our sample were alpha = .78 for the Positive Affect scale and = .74 for the Negative Affect scale.

##### 2.3.3.2 Patient Health Questionnaire-9

The PHQ-9 (62) is a 9-item self-administered depression screening scale that can be used to measure depression severity also based on the DSM-5 criteria (63). Items are rated on a 4-point Likert scale ranging from 0 = ‘Not at all’ to 3 = ‘Nearly every day.’ In this study, we investigated the frequency with which patients had experienced each of the nine symptoms of depression during the past 7 days. Higher scores indicate more severe depressive symptoms. Internal consistency in our sample for this measure was alpha = .86.

##### 2.3.3.3 Generalized Anxiety Disorder-7

The GAD-7 (64) is a 7-item self-report measure of the presence and severity of generalized anxiety disorder. Each item is scored 0 = ‘Not at all’ to 3 = ‘Nearly every day.’ We assessed the patient’s health status during the previous 7 days. Higher scores indicate more severe anxiety. Reliability in our study was alpha = .88.

##### 2.3.3.4 Single-item global measures of symptom severity, psychosocial functioning, and quality of life

The three single-item measures of symptom severity, psychosocial functioning, and quality of life developed by Zimmerman et al. (65) was adapted for this study. More specifically, the term ‘symptoms of depression’ was replaced by ‘symptoms for which you are in psychotherapeutic treatment.’ The item on symptom severity is rated on a 5-point Likert scale ranging from 0 = ‘None’ to 4 = ‘Severe.’ The psychosocial functioning item Likert scale ranges from 0 = ‘Not at all’ to 4 = ‘Extremely.’ The responses to the quality of life item range from 0 = ‘Very good, my life could hardly be better’ to 4 = ‘Very bad, my life could hardly be worse’.

#### 2.3.4 Therapeutic relationship domain

##### 2.3.4.1 in-Session Patient Affective Reactions Questionnaire

The SPARQ (10) is an 8-item patient-report measure of patterns of thought, feeling, and behavior activated and experienced in the therapeutic relationship. It is composed of two sub-scales of four items each: Positive Affect (delineates a secure and comfortable – from the patient’s perspective – experience of the therapeutic relationship) and Negative Affect (which is marked by items describing feelings of shyness and shame with the therapist, fear of speaking openly, worry of not being helped, and failure due to their need for help from the therapist). Patients rate on a 5-point Likert scale ranging from 0 = ‘Not at all true’ to 4 ‘Very true.’ Higher scores indicated greater emotional response. Initial validation of the SPARQ showed internal consistency of alpha = .86 and .74 respectively for the Positive and the Negative Affect scales. The SPARQ has been included as an Appendix.

##### 2.3.4.2 Working Alliance Inventory – Short Revised

The WAI-SR (66) is a 12-item self-report measure of the quality of the therapeutic alliance in the last session. It includes three subscales (four items each) that are: (a) agreement on the therapy’s tasks, (b) agreement on the therapy’s goals, and (c) development of a patient–therapist affective bond. Items are rated on a 6-point Likert scale ranging from 0 = ‘Not at all’ to 5 = ‘Completely.’ Higher scores indicate better alliance. Internal consistency in the current study was alpha = .95 for the total scale.

##### 2.3.4.3 Real Relationship Inventory-Client short form

The RRI-C-SF (67) is an 8-item self-report measure of perception of the strength of the real relationship, that is ‘the personal relationship existing between two or more people reflected in the degree to which each is genuine with the other and perceives and experiences the other in ways that befit the other’ (68). It consists of two subscales: Genuineness (i.e., ‘the ability to be who one truly is, as opposed to being phony or inauthentic’) and Realism (i.e., ‘perceiving experiencing or the other in ways that befit him or her [rather than projections based on fears and wishes related to significant others from the past]’) (68). Ratings are made on a 5-point Likert scale, ranging from 1 = ‘Strongly disagree’ to 5 ‘Strongly agree.’ Higher scores reflect stronger real relationships. In our internal consistency analysis, the RRI-C-SF showed alpha = .91 for the total scale.

##### 2.3.4.4 Post-Session Questionnaire

The part B of the PSQ (69) is a 4-single-item self-report measure of alliance ruptures and rupture resolution during the last therapy session. It consists of a gatekeeping-item exploring the occurrence (‘Yes’ or ‘No’) of any tension, conflict, problem, misunderstanding, or disagreement in the relationship with the therapist during the session, followed by three items assessing the highest degree of tension experienced (from 1 = ‘Low’ to 5 = ‘High’), the extent to which the problem was addressed in this session (from 1 = ‘Not at all’ to 5 = ‘Very much’), and the degree to which in the patient’s opinion the problem was resolved by the end of the session (from 1 = ‘Not at all’ to 5 = ‘Very much’).

#### 2.3.5 Session outcome domain

##### 2.3.5.1 Session Evaluation Scale

The SES (70) is a self-report 5-item subscale of the Helping Skills Measure (71) and assesses the patient’s perception of therapy session quality (which is a key aspect of the session outcome). Four items are rated on a 5-point Likert scale ranging from 1 = ‘Strongly disagree’ to 5 = ‘Strongly agree,’ while the fifth item scale ranges from 1 = ‘Very effective’ to 4 = ‘Ineffective.’ The score is obtained by summing the values of five items (after reversing the ones indicated) and then dividing by five. Higher scores indicate the perception of higher quality of the session. Reliability in our study was alpha = .86.

### 2.4 Data analysis

In the first step, Bartlett’s test of sphericity and Kaiser–Meyer–Olkin test were used to evaluate the suitability of the data for factor analysis. A confirmatory factor analysis (CFA) with robust maximum likelihood estimator was performed using the R package *lavaan* v0.6-12 (72) to test the fit of the two-factor model as identified in its validation study (10) – see the description in the Measure section. Multigroup CFA was performed using the R package *lavaan* to examine the measurement invariance of the SPARQ among patients who attended psychotherapy sessions in person (both face-to-face and on the couch) *versus* in remote mode (video and telephone calls). Furthermore, since the SPARQ was developed and preliminary validated using data from a sample completely composed of patients with psychiatric disorders, a multigroup CFA was conducted to examine the measurement invariance between patients with *versus* without a (self-reported) psychiatric diagnosis. In assessing CFA models fit, the following thresholds were considered: a comparative fit index (CFI) of .95 or greater, a Tucker Lewis index (TLI) of .95 or greater, a root mean square error of approximation (RMSEA) of .06 or less, and a standardized root mean square residual (SRMR) of .08 or less (73, 74). Graded response model (GRM) item response theory (IRT) models were employed using the R package *mirt* v1.37.1 (75) to get detailed information at the item level and to analyze option characteristics of the scale. To evaluate the psychometric properties of the SPARQ, the following statistical analyses were performed. Cronbach’s alpha and McDonald’s Omega total coefficients, as well as average inter-item *r*, were performed using the package *psych* v2.3.12 (76) to assess the internal consistency for each scale (49, 77). Correlations between subscales and (a) sociodemographic, clinical, and treatment variables and (b) validated measures of traits and state mental qualities of the patients, specific elements of the therapeutic relationship, and session outcome were calculated using the R package *correlation* v0.8.3 to provide criterion validity. Furthermore, pooled correlation matrices were averaged using Fisher’s *z*-transformation to produce an average inter-subtest correlation matrix for demographics, clinical (diagnoses), and treatment setting variables, as well as for traits, state, and therapeutic relationship measures scores. No missing data were present since the Qualtrics survey was set to force each response from the respondents. No missing data was encountered in the survey results, as the Qualtrics survey was configured to require respondents to answer each question, ensuring complete response data.

## 3 Results

### 3.1 Preliminary analyses

The Bartlett test of sphericity (*χ²*[45] = 2580, *p* < .001) and the Kaiser–Meyer–Olkin test (.87) supported data suitability for factor analysis.

### 3.2 Confirmatory factor analysis

**Figure 1:**
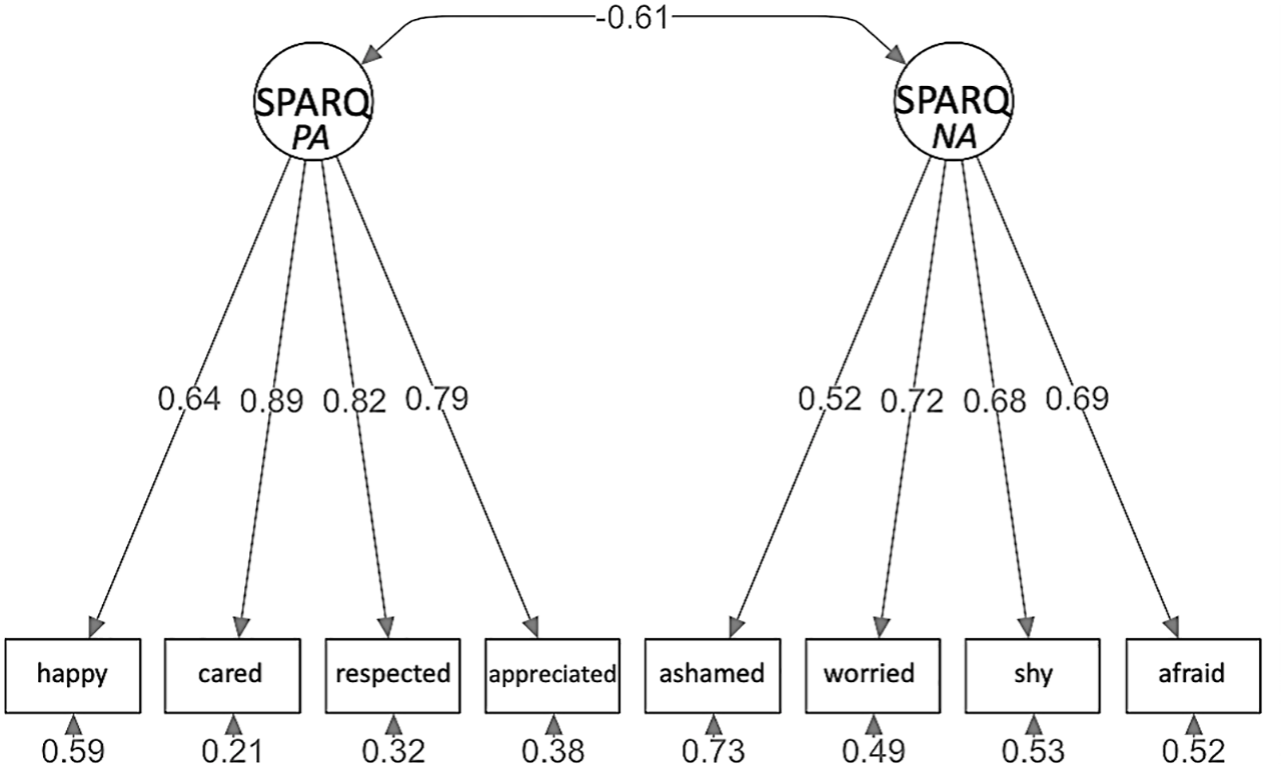
Measurement model from confirmatory factor analysis (*N* = 700) presenting fully standardized solution using robust maximum likelihood estimation. This figure presents abbreviated item content.

The two-factor model showed good fit to the data: χ²[19] = 5.62, CFI = .96, TLI = .94, RMSEA = .08 (90% CI [.07, .10]), and SRMR = .05 (see **Figure 1**). A bifactor model with a general factor of affective reaction and two specific factors of positive affect and negative affect demonstrated excellent indices of fit: χ²(*df* = 12) = 30.30, CFI = .99; TLI = .98; RMSEA = .05 (90% CI [.03, .07]); and SRMR = .02.

### 3.3 Invariance testing with multigroup CFA

Two multigroup CFA models were independently tested for measurement invariance between (a) patients attending in-person (either face-to-face or on the couch) and remote (via video or telephone call) sessions and (b) patients with or without a psychiatric diagnosis.

#### 3.3.1 Session format

The analysis of configural invariance demonstrated a robust fit of the two-factor model, as indicated by a CFI of .995 and a TLI of.993 for the free model. These values suggest a consistent factor structure across both groups, confirming that the pattern of item-factor loadings is uniform. The RMSEA was .063, and the SRMR was.058, further supporting the good fit of the model. See **Supplementary Material 1** for additional results.

Upon transition to metric invariance (weak model), there was a negligible shift in model fit. The ΔCFI was.000, indicating that there were no differences. The ΔTLI was +.001, moving from .993 to 0.994, and the ΔRMSEA was −.005, improving from .063 to .058. The ΔSRMR was +.002, reaching .060. These minimal changes suggest that the way items are weighted is largely consistent across groups.

Further analysis on scalar invariance (strong model) examined the equivalence of item intercepts across groups. In this phase, the model fit exhibited marginal alterations: ΔCFI remained virtually unchanged (+.001), resulting in a value of .996, while ΔTLI saw an increase of +.004 (from .993 to .997), and RMSEA improved, decreasing from .063 to .043. The SRMR value slightly increased reaching .060. These variations are not substantial.

The chi-square difference test comparing the weak and free models showed a nonsignificant difference (*p* = .640). Similarly, the comparison between the strong and free models did not show a significant difference (*p* = .480). These findings suggest that configural, metric, and scalar invariances are confirmed.

#### 3.3.2 Diagnostic status

Analysis of configural invariance demonstrated a robust fit, as indicated by a CFI of .993, a TLI of .989, a RMSEA of .074, and a SRMR of .063 for the unconstrained (free) model. These values suggest a consistent factor structure across both groups, confirming that the pattern of item-factor loadings is uniform.

Upon transition to metric invariance (weak model), there was a negligible shift in model fit. The ΔCFI was −.001, indicating a slight decrease from .993 to .992. The ΔTLI was +.001 (an increase rather than a decrease), moving from .989 to .990, and the ΔRMSEA remained constant at .000, maintaining the value of .074. The ΔSRMR also remained unchanged at .063. These minimal changes suggest that the way items are weighted is largely consistent across groups, although a deeper examination might be beneficial to confirm this observation. See **Supplementary Material 2** for additional results.

Further analysis on scalar invariance (strong model) examined the equivalence of item intercepts across groups. In this phase, the model fit exhibited marginal alterations: ΔCFI remained unchanged at .000, maintaining the value of .993, while ΔTLI saw a decrease of −.005 (from .989 to .984, not an increase to .994), and RMSEA improved, decreasing from .074 to .054. The SRMR saw a slight increase to .064. These changes suggest potential variations in the way items are interpreted between in-person and remote groups, although these variations are not substantial.

Finally, the chi-square difference tests comparing the free model with the weak and strong models provided significant insights. The comparison between the weak and free models was marginally significant (*p* = .057), suggesting a slight preference for the free model, while the comparison between the strong and free models did not show a significant difference (*p* = .360). In conclusion, while the analysis confirms configural invariance, it indicates less concern about metric and scalar invariance than initially anticipated. These findings suggest that the measurement and operationalization of constructs are largely similar between the two groups, with only minor differences that warrant further attention.

### 3.4 Item response theory

**Figure 2:**
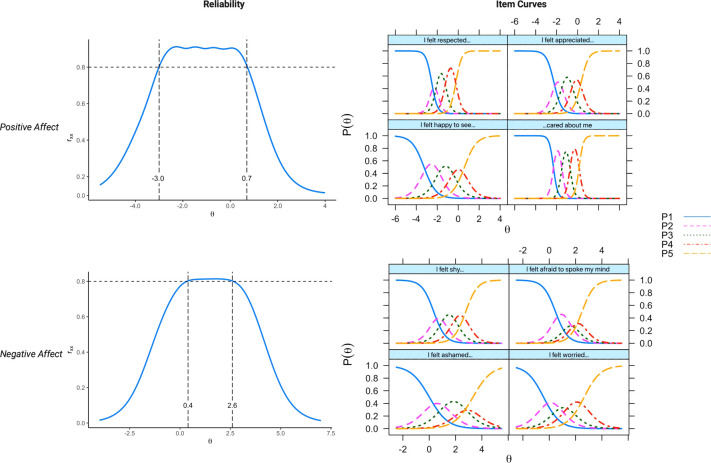
Item option characteristic curves and reliability for the scale scores. The reliability curves, represented in the range of 0 to 1.0, depict how reliability estimates vary across different levels of the underlying factor, specifically at low (negative θ values), average (θ = 0), and high (positive θ) levels. On the right, the curves illustrate the thresholds at which the probability of a patient selecting a higher response option on an item increases.

The items for the SPARQ scales were evaluated using Samejima’s graded response IRT model. For the Positive Affect scale, all four items demonstrated very high discrimination parameter values (>1.70). Similarly, the Negative Affect scale featured two items with high discrimination values (>1.35) and two with very high values (78). The Positive Affect scale demonstrated reliability >.80 from theta of −3.0 to +0.7. In contrast, the Negative Affect scale had reliability >.80 at theta ranging from +0.4 to +2.6. **Table 2** details the item discrimination and difficulty parameters. **Figure 2** shows the item characteristic curves and reliability for the scale scores.

**Table 2:** Item option characteristics for the three factors based on IRT models.

Scale	Item content	α	β1	β2	β3	β4
Positive Affect	I felt happy to see my therapist	1.74	–3.27	–1.85	–.54	.60
	I felt my therapist cared about me	4.93	–2.30	–1.50	–.71	.16
	I felt respected by my therapist	3.66	–2.53	–2.05	–1.22	–.22
	I felt appreciated by my therapist	2.79	–2.26	–1.47	–.52	.35
Negative Affect	I felt ashamed with my therapist about my fantasy, desires, mindset, behavior, or symptoms	1.43	.01	1.19	2.48	3.33
	I felt worried my therapist couldn’t help me	1.60	–.44	.66	1.53	2.65
	I felt shy, like I wanted to hide from my therapist or end the session early.	2.26	.34	1.07	1.94	2.77
	I felt afraid to spoke my mind, for fear of being judged, criticized, disliked by my therapist	2.17	.47	1.38	1.90	2.49

Graded response model for SPARQ scales.

### 3.5 Internal consistency and reliability

As can be seen in **Table 3**, both the Positive Affect (*k* = 4) and the Negative Affect (*k* = 4) scales were adequate in their internal consistency (49, 77) as measured by Cronbach alpha (.86 and .75, respectively), average inter-item *r* (.60 and .43, respectively), and McDonald omega total (.87 and .75, respectively).

**Table 3:** Descriptive statistics, internal consistency reliability, precision, and inter-scale correlations.

	Positive Affect	Negative Affect
Descriptive statistics		
Potential Range	0 to 16	0 to 16
Observed Range –	0 to 16	0 to 16
Mean, *SD*	12.26 (3.30)	3.18 (3.13)
POMP, *SD*	76.60 (20.60)	19.90 (19.50)
Skew	–.88	1.23
Kurtosis	–.34	1.23
Standard Error of Measurement (SE_m_)	1.19	1.57
Standard Error of Difference (SE_d_)	1.68	2.21
Internal consistency reliability		
Average inter-item *r*	.60	.43
Cronbach’s alpha	.86	.75
McDonald’s omega total	.87	.75
Clinical change benchmarks		
90% Critical Change	2.78	3.65
95% Critical Change	3.30	4.34
Minimally Important Difference (MID)	3.30	3.13
Jacobson benchmark threshold (5% tail)	LB: 5.79	UB: 9.31
Scale correlations		
SPARQ – Positive Affect	1	
SPARQ – Negative Affect	–.50^***^	1

LB, lower bound; POMP, percentage of maximum possible; UB, upper bound. MID was operationally defined as *d*, .5. *** *p* < .001, two-tailed.

### 3.6 Score precision

The mean scores on Positive Affect and Negative Affect scales were, respectively, 12.26 (*SD* = 3.30) and 3.18 (*SD* = 3.13).

**Table 3** reports the standard error of the measure, standard error of the difference, and reliable change indexes (79) as measures of score precision. Furthermore, clinical change benchmarks have been calculated. The 5^th^ percentile of the Positive Affect score was estimated to determine which score should be considered alarmingly low compared to the observed population distribution, and the 95^th^ percentile of the Negative Affect score to indicate which value should be considered alarmingly high. Lastly, estimates of the minimally important difference were provided as a criterion for what might be recognized as a meaningful shift in the patient’s affective tone.

### 3.7 Associations between SPARQ factors and sociodemographic, clinical, and treatment variables

Both the Positive and Negative Affect scales were very weakly correlated with the average correlation coefficients of demographics, clinical, and treatment variables. All individual correlations were very weak or weak, with *r*s ranging from −.20 to.20. Age, presence of any psychiatric disorder, and length of therapy were positively associated with the Positive Affect scale and negatively with the Negative Affect scale. **Table 4** presents all computed correlation coefficients.

**Table 4:** Criterion validity correlations with patient demographics, diagnoses, and objective therapy characteristics.

Criterion Variable	Positive Affect	Negative Affect
Age	.09^*^	–.20^***^
Biological sex	–.04	.07
Gender	–.05	.07
Education	.13^**^	–.07
4-year degree – High school graduate	2.15^* ^a^^	–1.04 ^a^
4-year degree – Some college	1.27^** ^a^^	–.79 ^a^
Ethnicity	–.02	.07
Average correlation matrix	.07	.10
Any psychiatric disorder	.14^**^	–.10
Any anxiety disorder	.06	.01
Any (unipolar) depressive disorder	.06	–.02
Any trauma- and stressor-related disorders	.08	–.01
Any neurodevelopmental disorder	.09	–.04
Any bipolar or related disorder	–.01	.00
Any eating disorder	.04	.06
Any disruptive behavior and dissocial disorder	–.02	.04
Schizophrenia or any other psychotic disorders	–.11^*^	.09
Any other psychiatric disorder	.00	–.04
Any cluster A personality disorder	.00	–.02
Any cluster B personality disorder	–.02	.14^**^
Any cluster C personality disorder	.04	.03
Average correlation matrix ^b^	.04	.04
Therapy length (months, ordinal; see prior table)	.20^***^	–.15^***^
Session frequency (ordinal, see prior table)	.12^**^	.05
Session attendance	.01	–.03
Therapy location	–.11^*^	.03
Private practice *vs.* Public health institution	1.27^* ^a^^	–.86 ^a^
Therapist’s sex	.02	.02
Average correlation matrix	.09	.06

Coefficients are point-biserial correlations for dichotomized variables, point-biserial correlations for dummy-coded categorical variables, Spearman correlations for ordinal variables, and Pearson correlations for continuous variables.

a ANOVA mean difference significant based on Tukey HSD post hoc correction. Positive values indicate that the scale ratings were significantly higher on average in the first group compared to the second.

b ‘Any psychiatric disorder’ excluded from the matrix.

*p < .05, **p < .01, ***p < .001

### 3.8 Criterion validity

The Positive Affect scale was very weakly correlated with the average correlation coefficients of both the trait (BFI-2-XS and LPFS-BF 2.0, mean absolute *r* = .13) and state (PHQ-9, GAD-7, and I-PANAS-SF, mean absolute *r* = .16) measures, whereas it was strongly (≥ .60) (80) correlated with the absolute average correlation coefficients of the therapeutic relationship measures. Looking more closely at the correlations (see **Table 5**), we can see that the Positive Affect scale was very strongly correlated with the WAI-SR total score and bond subscale and strongly with the WAI-SR goal and task subscales, the SES (session outcome measure), and the PSQ item on the extent to which the problem/conflict/misunderstanding experienced in the session was addressed in the same session. Furthermore, it was moderately correlated with the RRI-C total score and subscales, and the PSQ item about the degree to which the problem experienced in the session was resolved during the same encounter. Lastly, the Positive Affect scale was moderately and negatively correlated with the PSQ item on the magnitude of tension perceived by the subject in response to the problem that occurred during the session.

**Table 5:** Criterion validity correlations with validated scales.

Criterion Variable	Scale score *M (SD)*	Positive Affect	Negative Affect
Trait measures			
BFI-2-XS agreeableness	11.40 (2.39)	.20^***^	−.17^***^
BFI-2-XS conscientiousness	9.60 (3.01)	.03	−.10^*^
BFI-2-XS extraversion	8.21 (3.02)	.10^**^	−.16^***^
BFI-2-XS negative emotionality	10.90 (2.83)	−.04	.21^***^
BFI-2-XS open-mindedness	11.40 (2.46)	.08^*^	−.11^**^
LPFS-BF 2.0 total score	27.90 (7.32)	−.16^***^	.35^***^
LPFS-BF 2.0 self-functioning	15.20 (4.35)	−.12^**^	.32^***^
LPFS-BF 2.0 interpersonal functioning	12.70 (3.95)	−.17^***^	.29^***^
*Average correlation matrix* ^a^		.11	.20
State measures			
GAD-7	10.00 (6.10)	−.08^*^	.31^***^
I-PANAS-SF negative affect	11.60 (4.02)	−.15^***^	.40^***^
I-PANAS-SF positive affect	14.60 (4.14)	.22^***^	−.16^***^
PHQ-9	9.56 (6.24)	−.12^**^	.33^***^
SI – Psychosocial functioning	1.81 (1.07)	−.05	.24^***^
SI – Quality of life	1.79 (0.85)	−.15^***^	.23^***^
SI – Symptom severity	2.33 (0.92)	−.02	.18^***^
*Average correlation matrix*		.11	.27
Therapeutic relationship measures			
PSQ (yes) *% (F)*	16.00% (112)	−.28^***^	.33^***^
Degree of tension^b^	2.75 (1.04)	−.40^***^	.37^***^
Extent issue addressed^c^	2.90 (1.34)	.61^***^	−.30^**^
Degree of resolution^d^	2.73 (1.49)	.55^***^	−.36^***^
RRI-C-SF total score	31.50 (6.20)	.52^***^	−.40^***^
RRI-C-SF genuineness	16.20 (3.73)	.56^***^	−.43^***^
RRI-C-SF realism	15.30 (3.37)	.58^***^	−.41^***^
WAI-SR total score	40.50 (13.10)	.80^***^	−.48^***^
WAI-SR goal	13.60 (4.93)	.68^***^	−.42^***^
WAI-SR task	12.50 (4.80)	.67^***^	−.44^***^
WAI-SR bond	14.50 (4.61)	.83^***^	−.46^***^
*Average correlation matrix* ^e^		.61	.40
Session outcome measure			
SES	4.06 (0.84)	.66^***^	−.50^***^

BFI-2-XS, Big Five Inventory–2-Extra-Short form; GAD-7, Generalized Anxiety Disorder-7; I-PANAS-SF, International Positive and Negative Affect Schedule - Short Form; LPFS, Level of Personality Functioning Scale; PHQ-9, Patient Health Questionnaire-9; PSQ, Single-item indices of ruptures and rupture resolution of the Post-Session Questionnaire; RRI-C, Real Relationship Inventory–Client form; SES, Session Evaluation Scale; SI, Single-item global measures of symptom severity, psychosocial functioning, and quality of life; WAI-SR, Working Alliance Inventory – Short Revised.

a LPFS-BF 2.0 total score excluded from the matrix.

b Highest degree of tension felt during the session.

c Extent to which the problem was addressed in the session.

d Degree to which the problem was resolved by the end of the session.

e PSQ (yes), RRI-C total score, and WAI-SR total score excluded from the matrix.

The Negative Affect scale was weakly correlated with the average correlation coefficients of both the trait and state measures, and moderately correlated with the average correlation coefficients of the therapeutic relationship measures. Looking at the individual correlations, we can see that the Negative Affect scale had no strong correlations, but was moderately correlated with the SES, the WAI-SR total score and subscales, and the RRI-C total score and subscales. Furthermore, it was moderately correlated with the PSQ item on the magnitude of tension experienced by the patient. The correlations between the SPARQ scales and all the measures of patients’ personality characteristics and current mental health state were weak (*r* ≤ .39) or very weak (*r* ≤ .19). **Table 5** presents all computed correlation coefficients.

## 4 Discussion

In this study, our objective was to examine the reliability and validity of a new instrument intended to gather potentially clinically helpful information about affective reactions in the adult patient during individual psychotherapy sessions, the SPARQ. We evaluated this scale using factor analyses and item response theory.

Patient feedback (self- or other) feedback, despite its inherent biases and distortions (81, 82), is an important indicator of their inner experience during psychotherapy sessions. Conscious emotional processes, as reflected in such feedback, play a crucial role in enhancing therapy outcomes (83) and understanding the reasons for early termination of therapy (84). Additionally, patient-reported information provides an essential balance to therapist assessments, which may be skewed by biases in evaluating patient emotions and understanding. In fact, these biases contribute to approximately 30% of the variance in therapist ratings, even after considering perceived emotional intelligence (82). By incorporating patients’ views on their emotional responses, therapists gain access to what could be considered ‘objective data’ from the patient’s perspective. This approach not only provides more comprehensive information, but also helps to challenge negative interpersonal perceptions, foster deeper understanding, and strengthen the therapeutic alliance and outcomes. Studies on routine outcome monitoring in psychotherapy suggest that paying attention to patients’ emotional responses towards their therapists can be particularly beneficial for those struggling with therapy (85–87). Furthermore, the practice of psychological assessment, when coupled with tailored feedback, can serve as a therapeutic tool in itself, producing significant positive effects on treatment processes (88). The SPARQ could signify an advanced progression in the field of measurement feedback systems, which are based on the employment of valid, reliable, and standardized methods to improve mental health treatment outcomes (89).

The two dimensions of the SPARQ reflect the emotional patterns that typically emerge in the context of psychotherapeutic practice (90–93). This alignment enables therapists and researchers to effectively discern and monitor patient emotional responses towards their therapists. Additionally, it facilitates the measurement of these emotional responses over various sessions and examines their connection with both the individual session and the overall treatment outcomes. The utility of the SPARQ could potentially extend beyond mere routine monitoring, becoming instrumental in transference work (86). This involves exploring how patients engage with their therapists, thereby improving the therapist’s understanding of the types and intensities of emotional reactions encountered. The dimensions identified in the SPARQ likely represent a composite of the intrinsic interpersonal dynamics of the patient, which is evoked in part by the therapist and the therapeutic environment, as well as the dynamics arising from the interactive attitudes and behaviors of both the patient and the therapist during sessions.

The SPARQ showed very good reliability and excellent model fit indices. The average inter-item correlation for the Positive Affect scale was.60, indicating a strong correlation among items. However, in this specific case, i.e., a scale with a very narrow focus, the elevated inter-item correlation results from the assessment of different but closely interrelated facets of the construct being assessed.

Importantly, our findings reveal that the SPARQ maintains measurement invariance whether applied in in-person or remote psychotherapeutic settings. This indicates that SPARQ is an effective tool to uniformly assess patients’ affective reactions in both telepsychotherapy and conventional face-to-face psychotherapy. The significance of this result is enhanced by the rapid shift of teletherapy from a complementary option to becoming a mainstream treatment method in recent years (94), a transition that has been markedly accelerated by the COVID-19 pandemic (95, 96).

Evidence of convergence validity was provided by, on the one hand, very weak correlations of the SPARQ scales with patient demographic, clinical, and treatment variables, as well as weak correlations with traits and state measures, and, on the other hand, moderate to strong correlations with validated measures of specific elements of the therapeutic relationship and a measure of session outcome. All findings of the differential pattern of correlations support the assumption that the affects assessed by our scales are specific to the therapy session and appear to be influenced to a small to moderate degree by the patient’s general psychopathology or extra-therapeutic factors.

However, we found that the negative affective pattern was not completely arbitrary but tended to relate to both the presence of a cluster B personality disorder and the severity of personality pathology. This trend is clinically meaningful and predictable based on the existing literature. In fact, previous studies showed that personality disorders are related to the negative dimensions of the therapeutic relationship (43, 97, 98). These results suggest that therapists treating a patient with a personality disorder, notably cluster B personality disorders, can expect the occurrence of negative attitudes and behavior. By being aware of this situation, the therapist may be able to provide a prompt and effective therapeutic intervention that, among other things, can help reduce premature discontinuation [which is a particularly high risk in patients with a personality disorder (99)].

As expected, the Positive Affect was positively correlated with the measures of working alliance, alliance ruptures and reparations (more specifically, the extent to which the rupture was addressed and resolved in the session), real relationship, and session outcome. Predictability, it was more strongly correlated with the bond component of the WAI-SR than with the other two components. Regarding the Negative Affect, as expected, it was negatively and moderately correlated with the measures of working alliance, real relationship, and session outcome, and positively though weakly correlated with the occurrence of alliance ruptures.

Regarding the relationship between alliance and patient affective reactions toward their therapist, evidence shows that affective reactions predict a substantial part of alliance throughout the course of psychotherapeutic treatment (44). On a clinical level, these findings suggest that by paying adequate attention early in treatment to patterns of affective reactions, therapists can become aware of the potential risks to the alliance relevant to a specific patient, and learn more about the potential benefits of its formation. Ruptures in the alliance, which predict worse treatment outcomes when remaining unrepaired (47), may originate from conflictual emotional processes that emerge in the in-session relational patterns of patients (44, 100). A careful exploration of patterns of affective reactions toward the therapist and alliance ruptures may place the therapist in the best position to manage the alliance by promptly using interventions aimed at disproving negative interpersonal expectations. Empathically tuned interpretation of their in-session affective reactions toward the therapist may be particularly useful in improving patients’ affective awareness and insight into the maladaptive emotional and interpersonal patterns (101, 102) and in jointly exploring in-session interactions (103, 104).

Concerning the session outcome, it is important to note that studies on the relationship between affective reactions toward the therapist and session/treatment outcome have produced contradictory results. As regards negative affective reactions, most of the studies found that the amount of negative transference is negatively related to session outcome (105, 106) and symptom change (107). However, another study found no associations (108). Regarding positive affective reactions, although one study found a positive relationship between positive transference and outcome (106), numerous other studies found no associations (105, 107, 109). These inconsistencies can be partially explained by Gelso and Carter’s (110) theorization that gain in patient insight moderates the effect of transference on the outcome, especially about insight with regard to their negative responses. Two studies offer empirical support, indicating that when patient insight is high, negative reactions relates positively to session and treatment outcomes, whereas in combined with low levels of insight, negative transference harms treatment (108, 111). Furthermore, a meta-analysis on the association between insight and outcome of psychotherapy found a significant moderate relationship (*r* = .31) (112).

The above allows us to believe that (a) the SPARQ is a clean measure of the in-session therapeutic relationship, (b) the construct measured by the Positive Affect scale is similar, but not isomorphic, to that of the bond alliance, (c) the construct measured by the Negative Affect scale is different with respect to that of the working alliance. Furthermore, our results prompt us to consider whether the positive affect experienced by the patient toward the therapist can foster the working alliance, especially the bond part of it. We also hypothesize that the Positive Affect can act as a buffer against the negative consequences of the (co)presence of the Negative Affect on the strength of the therapeutic relationship.

### 4.1 Limitations

The findings from this study need to be interpreted in light of some limitations. First, our data are based exclusively on the patient’s perspective. Although patients’ perceptions are fundamental within the psychotherapeutic treatment, they present only one element of a complex dynamic system. This limitation includes the potential bias in self-reporting one’s own affective reactions versus recording respondent’s physiological responses (81), which can capture aspects of emotional reactions that are beyond respondents’ consciousness. However, self-report measures contribute important information on patients’ perceptions and internal experiences of their therapist during a session. Assessing and considering the conscious emotional experience of the patient is clinically crucial when choosing the most appropriate therapeutic intervention. Second, our study lacks measurements of patients’ awareness of mental states in themselves (i.e., mentalization) and capacity to identify and verbalize emotional states (i.e., alexithymia), as well as suppressive emotion regulation strategies, which may be important for collecting valid and reliable data on in-session emotional reactions. This limitation is strictly related to the first one, because here too is there the opportunity for integrating self-rated questionnaires with observer-rated methods (113, 114). A third limitation is that no information on what type of psychotherapy participants were receiving has been collected.

### 4.2 Future directions

Future research using the SPARQ should examine the affective states and processes assessed from multiple perspectives to further test their validity, assess the correlates, and understand how the scores relate to the therapist’s perceptions of these phenomena, as well as the therapist’s own affective reactions toward the patient. Longitudinal research is required to investigate how these phenomena unfold over the course of psychotherapy and predict different trajectories and outcomes. Lastly, research is needed to evaluate whether the SPARQ can serve as an efficient tool for routine monitoring and systematic client feedback. Currently, it is being tested in a randomized controlled trial to determine whether its use as part of a brief postsession battery can improve the self-monitoring and reflection of patients about their emotional reactions towards their psychotherapists (115), with the ultimate goal of determining if such self-monitoring and reflection can lead to improvements in the quality of the therapeutic relationship, specifically in terms of the working alliance and the real relationship, and to improve treatment outcomes.

## 5 Conclusion

Our findings support the use of the SPARQ in both clinical and research settings, with particular value for assessing the patient’s subjective affective reactions towards their therapist and session-level affective processes.

## Data availability statement

Following the publication of all the research findings, the data will be publicly shared through the Open Science Framework (https://osf.io/amzqk/), further inquiries can be directed to the corresponding author/s.

## Ethics statement

The studies involving humans were approved by Institutional Review Board (IRB) of the University of North Carolina at Chapel Hill (Study #: 23-0216; Approval Date: 3/06/2023). The studies were conducted in accordance with the local legislation and institutional requirements. The participants provided their written informed consent to participate in this study.

## Author contributions

AS: Conceptualization, Data curation, Formal analysis, Funding acquisition, Investigation, Methodology, Project administration, Resources, Software, Validation, Visualization, Writing – original draft, Writing – review & editing. PF-P: Supervision, Writing – review & editing. EV: Funding acquisition, Supervision, Writing – review & editing. EY: Conceptualization, Funding acquisition, Methodology, Resources, Supervision, Writing – review & editing.

## Appendix

*in-Session Patient Affective Reactions Questionnaire (SPARQ)*

The following is a series of statements that people in psychotherapy might use to describe how they feel toward their psychotherapist. Think about your last psychotherapy session and remember some details from it. Then read each statement and indicate how you felt during that session. Select the response that corresponds with your answer with ‘0’ being not at all and ‘4’ being very much. Do not worry if your responses appear to be inconsistent, as people often experience mixed and conflicting feelings.

**Table:**

Item nr.		Not at all	A little	Somewhat	A lot	Very much
1	I felt happy to see my therapist.	0	1	2	3	4
2	I felt ashamed with my therapist about my fantasy, desires, mindset, behavior, or symptoms.	0	1	2	3	4
3	I felt worried my therapist couldn’t help me.	0	1	2	3	4
4	I felt shy, like I wanted to hide from my therapist or end the session early.	0	1	2	3	4
5	I felt afraid to spoke my mind, for fear of being judged, criticized, disliked by my therapist.	0	1	2	3	4
6	I felt my therapist cared about me.	0	1	2	3	4
7	I felt respected by my therapist.	0	1	2	3	4
8	I felt appreciated by my therapist.	0	1	2	3	4

Positive Affect items: 1, 6, 7, and 8. Negative Affect items: 2, 3, 4, and 5.
