# Supplementary material for: Assessing the patient’s affective perception of their psychotherapist: validation of the in-Session Patient Affective Reactions Questionnaire
Source: Front Psychiatry. 2024 Apr 24;15:1346760. doi: 10.3389/fpsyt.2024.1346760 (PMC11096799; doi:10.3389/fpsyt.2024.1346760)
Supplement: Supplementary file 1 [file DataSheet_1.docx]

**Supplementary Table 1.** Summary of Standardized Estimates.

| lhs | op | rhs | block | group | est | se | z | p-value | ci.lower | ci.upper | std.lv | std.all | std.nox |
| --- | --- | --- | --- | --- | --- | --- | --- | --- | --- | --- | --- | --- | --- |
| pos | =~ | sparq1 | 1 | 1 | 0.7346 | 0.0289 | 25.397 | < 0.001 | 0.6779 | 0.7913 | 0.7346 | 0.7346 | 0.7346 |
| pos | =~ | sparq7 | 1 | 1 | 0.9274 | 0.0143 | 64.808 | < 0.001 | 0.8993 | 0.9554 | 0.9274 | 0.9274 | 0.9274 |
| pos | =~ | sparq8 | 1 | 1 | 0.8951 | 0.0182 | 49.126 | < 0.001 | 0.8594 | 0.9309 | 0.8951 | 0.8951 | 0.8951 |
| pos | =~ | sparq9 | 1 | 1 | 0.8642 | 0.0191 | 45.189 | < 0.001 | 0.8268 | 0.9017 | 0.8642 | 0.8642 | 0.8642 |
| neg | =~ | sparq2 | 1 | 1 | 0.5122 | 0.0466 | 11.002 | < 0.001 | 0.4210 | 0.6035 | 0.5122 | 0.5122 | 0.5122 |
| neg | =~ | sparq3 | 1 | 1 | 0.8089 | 0.0346 | 23.347 | < 0.001 | 0.7410 | 0.8768 | 0.8089 | 0.8089 | 0.8089 |
| neg | =~ | sparq4 | 1 | 1 | 0.7216 | 0.0401 | 17.993 | < 0.001 | 0.6430 | 0.8003 | 0.7216 | 0.7216 | 0.7216 |
| neg | =~ | sparq6 | 1 | 1 | 0.7606 | 0.0430 | 17.697 | < 0.001 | 0.6764 | 0.8449 | 0.7606 | 0.7606 | 0.7606 |
| sparq1 | \| | t1 | 1 | 1 | -2.1968 | 0.1593 | -13.791 | < 0.001 | -2.5090 | -1.8846 | -2.1968 | -2.1968 | -2.1968 |
| sparq1 | \| | t2 | 1 | 1 | -1.3059 | 0.0838 | -15.588 | < 0.001 | -1.4701 | -1.1417 | -1.3059 | -1.3059 | -1.3059 |
| sparq1 | \| | t3 | 1 | 1 | -0.3341 | 0.0619 | -5.396 | < 0.001 | -0.4554 | -0.2127 | -0.3341 | -0.3341 | -0.3341 |
| sparq1 | \| | t4 | 1 | 1 | 0.4415 | 0.0629 | 7.022 | < 0.001 | 0.3182 | 0.5647 | 0.4415 | 0.4415 | 0.4415 |
| sparq7 | \| | t1 | 1 | 1 | -2.0330 | 0.1375 | -14.788 | < 0.001 | -2.3024 | -1.7635 | -2.0330 | -2.0330 | -2.0330 |
| sparq7 | \| | t2 | 1 | 1 | -1.4089 | 0.0885 | -15.916 | < 0.001 | -1.5823 | -1.2354 | -1.4089 | -1.4089 | -1.4089 |
| sparq7 | \| | t3 | 1 | 1 | -0.7117 | 0.0666 | -10.687 | < 0.001 | -0.8423 | -0.5812 | -0.7117 | -0.7117 | -0.7117 |
| sparq7 | \| | t4 | 1 | 1 | 0.1351 | 0.0609 | 2.220 | < 0.001 | 0.0158 | 0.2544 | 0.1351 | 0.1351 | 0.1351 |
| sparq8 | \| | t1 | 1 | 1 | -2.1968 | 0.1593 | -13.791 | < 0.001 | -2.5090 | -1.8846 | -2.1968 | -2.1968 | -2.1968 |
| sparq8 | \| | t2 | 1 | 1 | -1.7819 | 0.1126 | -15.825 | < 0.001 | -2.0026 | -1.5612 | -1.7819 | -1.7819 | -1.7819 |
| sparq8 | \| | t3 | 1 | 1 | -1.1224 | 0.0768 | -14.611 | < 0.001 | -1.2730 | -0.9719 | -1.1224 | -1.1224 | -1.1224 |
| sparq8 | \| | t4 | 1 | 1 | -0.2064 | 0.0611 | -3.376 | < 0.001 | -0.3263 | -0.0866 | -0.2064 | -0.2064 | -0.2064 |
| sparq9 | \| | t1 | 1 | 1 | -1.8753 | 0.1208 | -15.519 | < 0.001 | -2.1122 | -1.6385 | -1.8753 | -1.8753 | -1.8753 |
| sparq9 | \| | t2 | 1 | 1 | -1.1910 | 0.0792 | -15.035 | < 0.001 | -1.3463 | -1.0358 | -1.1910 | -1.1910 | -1.1910 |
| sparq9 | \| | t3 | 1 | 1 | -0.3841 | 0.0623 | -6.162 | < 0.001 | -0.5062 | -0.2619 | -0.3841 | -0.3841 | -0.3841 |
| sparq9 | \| | t4 | 1 | 1 | 0.2667 | 0.0615 | 4.339 | < 0.001 | 0.1462 | 0.3871 | 0.2667 | 0.2667 | 0.2667 |
| sparq2 | \| | t1 | 1 | 1 | 0.0527 | 0.0607 | 0.869 | < 0.001 | -0.0662 | 0.1717 | 0.0527 | 0.0527 | 0.0527 |
| sparq2 | \| | t2 | 1 | 1 | 0.8300 | 0.0689 | 12.045 | < 0.001 | 0.6949 | 0.9651 | 0.8300 | 0.8300 | 0.8300 |
| sparq2 | \| | t3 | 1 | 1 | 1.6540 | 0.1029 | 16.071 | < 0.001 | 1.4523 | 1.8557 | 1.6540 | 1.6540 | 1.6540 |
| sparq2 | \| | t4 | 1 | 1 | 2.1356 | 0.1505 | 14.188 | < 0.001 | 1.8406 | 2.4307 | 2.1356 | 2.1356 | 2.1356 |
| sparq3 | \| | t1 | 1 | 1 | -0.3590 | 0.0621 | -5.779 | < 0.001 | -0.4807 | -0.2372 | -0.3590 | -0.3590 | -0.3590 |
| sparq3 | \| | t2 | 1 | 1 | 0.4937 | 0.0634 | 7.783 | < 0.001 | 0.3694 | 0.6181 | 0.4937 | 0.4937 | 0.4937 |
| sparq3 | \| | t3 | 1 | 1 | 1.0284 | 0.0739 | 13.917 | < 0.001 | 0.8836 | 1.1733 | 1.0284 | 1.0284 | 1.0284 |
| sparq3 | \| | t4 | 1 | 1 | 1.7018 | 0.1063 | 16.004 | < 0.001 | 1.4934 | 1.9103 | 1.7018 | 1.7018 | 1.7018 |
| sparq4 | \| | t1 | 1 | 1 | 0.3094 | 0.0617 | 5.012 | < 0.001 | 0.1884 | 0.4304 | 0.3094 | 0.3094 | 0.3094 |
| sparq4 | \| | t2 | 1 | 1 | 0.8466 | 0.0693 | 12.222 | < 0.001 | 0.7109 | 0.9824 | 0.8466 | 0.8466 | 0.8466 |
| sparq4 | \| | t3 | 1 | 1 | 1.5294 | 0.0950 | 16.099 | < 0.001 | 1.3432 | 1.7156 | 1.5294 | 1.5294 | 1.5294 |
| sparq4 | \| | t4 | 1 | 1 | 2.0816 | 0.1434 | 14.514 | < 0.001 | 1.8005 | 2.3626 | 2.0816 | 2.0816 | 2.0816 |
| sparq6 | \| | t1 | 1 | 1 | 0.4158 | 0.0626 | 6.641 | < 0.001 | 0.2931 | 0.5385 | 0.4158 | 0.4158 | 0.4158 |
| sparq6 | \| | t2 | 1 | 1 | 1.1675 | 0.0784 | 14.898 | < 0.001 | 1.0139 | 1.3211 | 1.1675 | 1.1675 | 1.1675 |
| sparq6 | \| | t3 | 1 | 1 | 1.5294 | 0.0950 | 16.099 | < 0.001 | 1.3432 | 1.7156 | 1.5294 | 1.5294 | 1.5294 |
| sparq6 | \| | t4 | 1 | 1 | 1.9105 | 0.1242 | 15.378 | < 0.001 | 1.6670 | 2.1539 | 1.9105 | 1.9105 | 1.9105 |
| sparq1 | ~~ | sparq1 | 1 | 1 | 0.4603 | 0.0000 | NA | NA | 0.4603 | 0.4603 | 0.4603 | 0.4603 | 0.4603 |
| sparq7 | ~~ | sparq7 | 1 | 1 | 0.1400 | 0.0000 | NA | NA | 0.1400 | 0.1400 | 0.1400 | 0.1400 | 0.1400 |
| sparq8 | ~~ | sparq8 | 1 | 1 | 0.1987 | 0.0000 | NA | NA | 0.1987 | 0.1987 | 0.1987 | 0.1987 | 0.1987 |
| sparq9 | ~~ | sparq9 | 1 | 1 | 0.2531 | 0.0000 | NA | NA | 0.2531 | 0.2531 | 0.2531 | 0.2531 | 0.2531 |
| sparq2 | ~~ | sparq2 | 1 | 1 | 0.7376 | 0.0000 | NA | NA | 0.7376 | 0.7376 | 0.7376 | 0.7376 | 0.7376 |
| sparq3 | ~~ | sparq3 | 1 | 1 | 0.3457 | 0.0000 | NA | NA | 0.3457 | 0.3457 | 0.3457 | 0.3457 | 0.3457 |
| sparq4 | ~~ | sparq4 | 1 | 1 | 0.4792 | 0.0000 | NA | NA | 0.4792 | 0.4792 | 0.4792 | 0.4792 | 0.4792 |
| sparq6 | ~~ | sparq6 | 1 | 1 | 0.4215 | 0.0000 | NA | NA | 0.4215 | 0.4215 | 0.4215 | 0.4215 | 0.4215 |
| pos | ~~ | pos | 1 | 1 | 1.0000 | 0.0000 | NA | NA | 1.0000 | 1.0000 | 1.0000 | 1.0000 | 1.0000 |
| neg | ~~ | neg | 1 | 1 | 1.0000 | 0.0000 | NA | NA | 1.0000 | 1.0000 | 1.0000 | 1.0000 | 1.0000 |
| pos | ~~ | neg | 1 | 1 | -0.6760 | 0.0361 | -18.741 | < 0.001 | -0.7467 | -0.6053 | -0.6760 | -0.6760 | -0.6760 |
| sparq1 | ~*~ | sparq1 | 1 | 1 | 1.0000 | 0.0000 | NA | NA | 1.0000 | 1.0000 | 1.0000 | 1.0000 | 1.0000 |
| sparq7 | ~*~ | sparq7 | 1 | 1 | 1.0000 | 0.0000 | NA | NA | 1.0000 | 1.0000 | 1.0000 | 1.0000 | 1.0000 |
| sparq8 | ~*~ | sparq8 | 1 | 1 | 1.0000 | 0.0000 | NA | NA | 1.0000 | 1.0000 | 1.0000 | 1.0000 | 1.0000 |
| sparq9 | ~*~ | sparq9 | 1 | 1 | 1.0000 | 0.0000 | NA | NA | 1.0000 | 1.0000 | 1.0000 | 1.0000 | 1.0000 |
| sparq2 | ~*~ | sparq2 | 1 | 1 | 1.0000 | 0.0000 | NA | NA | 1.0000 | 1.0000 | 1.0000 | 1.0000 | 1.0000 |
| sparq3 | ~*~ | sparq3 | 1 | 1 | 1.0000 | 0.0000 | NA | NA | 1.0000 | 1.0000 | 1.0000 | 1.0000 | 1.0000 |
| sparq4 | ~*~ | sparq4 | 1 | 1 | 1.0000 | 0.0000 | NA | NA | 1.0000 | 1.0000 | 1.0000 | 1.0000 | 1.0000 |
| sparq6 | ~*~ | sparq6 | 1 | 1 | 1.0000 | 0.0000 | NA | NA | 1.0000 | 1.0000 | 1.0000 | 1.0000 | 1.0000 |
| sparq1 | ~1 |  | 1 | 1 | 0.0000 | 0.0000 | NA | NA | 0.0000 | 0.0000 | 0.0000 | 0.0000 | 0.0000 |
| sparq7 | ~1 |  | 1 | 1 | 0.0000 | 0.0000 | NA | NA | 0.0000 | 0.0000 | 0.0000 | 0.0000 | 0.0000 |
| sparq8 | ~1 |  | 1 | 1 | 0.0000 | 0.0000 | NA | NA | 0.0000 | 0.0000 | 0.0000 | 0.0000 | 0.0000 |
| sparq9 | ~1 |  | 1 | 1 | 0.0000 | 0.0000 | NA | NA | 0.0000 | 0.0000 | 0.0000 | 0.0000 | 0.0000 |
| sparq2 | ~1 |  | 1 | 1 | 0.0000 | 0.0000 | NA | NA | 0.0000 | 0.0000 | 0.0000 | 0.0000 | 0.0000 |
| sparq3 | ~1 |  | 1 | 1 | 0.0000 | 0.0000 | NA | NA | 0.0000 | 0.0000 | 0.0000 | 0.0000 | 0.0000 |
| sparq4 | ~1 |  | 1 | 1 | 0.0000 | 0.0000 | NA | NA | 0.0000 | 0.0000 | 0.0000 | 0.0000 | 0.0000 |
| sparq6 | ~1 |  | 1 | 1 | 0.0000 | 0.0000 | NA | NA | 0.0000 | 0.0000 | 0.0000 | 0.0000 | 0.0000 |
| pos | ~1 |  | 1 | 1 | 0.0000 | 0.0000 | NA | NA | 0.0000 | 0.0000 | 0.0000 | 0.0000 | 0.0000 |
| neg | ~1 |  | 1 | 1 | 0.0000 | 0.0000 | NA | NA | 0.0000 | 0.0000 | 0.0000 | 0.0000 | 0.0000 |
| pos | =~ | sparq1 | 2 | 2 | 0.6937 | 0.0382 | 18.142 | < 0.001 | 0.6188 | 0.7687 | 0.6937 | 0.6937 | 0.6937 |
| pos | =~ | sparq7 | 2 | 2 | 0.9069 | 0.0218 | 41.574 | < 0.001 | 0.8642 | 0.9497 | 0.9069 | 0.9069 | 0.9069 |
| pos | =~ | sparq8 | 2 | 2 | 0.9033 | 0.0235 | 38.512 | < 0.001 | 0.8573 | 0.9493 | 0.9033 | 0.9033 | 0.9033 |
| pos | =~ | sparq9 | 2 | 2 | 0.7914 | 0.0315 | 25.135 | < 0.001 | 0.7297 | 0.8531 | 0.7914 | 0.7914 | 0.7914 |
| neg | =~ | sparq2 | 2 | 2 | 0.5953 | 0.0515 | 11.565 | < 0.001 | 0.4944 | 0.6962 | 0.5953 | 0.5953 | 0.5953 |
| neg | =~ | sparq3 | 2 | 2 | 0.8088 | 0.0440 | 18.388 | < 0.001 | 0.7226 | 0.8950 | 0.8088 | 0.8088 | 0.8088 |
| neg | =~ | sparq4 | 2 | 2 | 0.7440 | 0.0462 | 16.088 | < 0.001 | 0.6533 | 0.8346 | 0.7440 | 0.7440 | 0.7440 |
| neg | =~ | sparq6 | 2 | 2 | 0.7978 | 0.0466 | 17.119 | < 0.001 | 0.7065 | 0.8892 | 0.7978 | 0.7978 | 0.7978 |
| sparq1 | \| | t1 | 2 | 2 | -2.2894 | 0.2185 | -10.479 | < 0.001 | -2.7175 | -1.8612 | -2.2894 | -2.2894 | -2.2894 |
| sparq1 | \| | t2 | 2 | 2 | -1.3071 | 0.1052 | -12.431 | < 0.001 | -1.5132 | -1.1010 | -1.3071 | -1.3071 | -1.3071 |
| sparq1 | \| | t3 | 2 | 2 | -0.4375 | 0.0788 | -5.551 | < 0.001 | -0.5919 | -0.2830 | -0.4375 | -0.4375 | -0.4375 |
| sparq1 | \| | t4 | 2 | 2 | 0.4579 | 0.0791 | 5.790 | < 0.001 | 0.3029 | 0.6128 | 0.4579 | 0.4579 | 0.4579 |
| sparq7 | \| | t1 | 2 | 2 | -2.4395 | 0.2549 | -9.570 | < 0.001 | -2.9391 | -1.9399 | -2.4395 | -2.4395 | -2.4395 |
| sparq7 | \| | t2 | 2 | 2 | -1.4241 | 0.1120 | -12.716 | < 0.001 | -1.6436 | -1.2046 | -1.4241 | -1.4241 | -1.4241 |
| sparq7 | \| | t3 | 2 | 2 | -0.6289 | 0.0818 | -7.685 | < 0.001 | -0.7893 | -0.4685 | -0.6289 | -0.6289 | -0.6289 |
| sparq7 | \| | t4 | 2 | 2 | 0.2042 | 0.0767 | 2.662 | < 0.001 | 0.0539 | 0.3544 | 0.2042 | 0.2042 | 0.2042 |
| sparq8 | \| | t1 | 2 | 2 | -2.4395 | 0.2549 | -9.570 | < 0.001 | -2.9391 | -1.9399 | -2.4395 | -2.4395 | -2.4395 |
| sparq8 | \| | t2 | 2 | 2 | -1.9475 | 0.1606 | -12.129 | < 0.001 | -2.2622 | -1.6328 | -1.9475 | -1.9475 | -1.9475 |
| sparq8 | \| | t3 | 2 | 2 | -1.1327 | 0.0968 | -11.702 | < 0.001 | -1.3224 | -0.9430 | -1.1327 | -1.1327 | -1.1327 |
| sparq8 | \| | t4 | 2 | 2 | -0.1760 | 0.0765 | -2.300 | < 0.001 | -0.3260 | -0.0260 | -0.1760 | -0.1760 | -0.1760 |
| sparq9 | \| | t1 | 2 | 2 | -1.9475 | 0.1606 | -12.129 | < 0.001 | -2.2622 | -1.6328 | -1.9475 | -1.9475 | -1.9475 |
| sparq9 | \| | t2 | 2 | 2 | -1.3291 | 0.1064 | -12.497 | < 0.001 | -1.5375 | -1.1206 | -1.3291 | -1.3291 | -1.3291 |
| sparq9 | \| | t3 | 2 | 2 | -0.4784 | 0.0794 | -6.029 | < 0.001 | -0.6339 | -0.3229 | -0.4784 | -0.4784 | -0.4784 |
| sparq9 | \| | t4 | 2 | 2 | 0.3973 | 0.0783 | 5.072 | < 0.001 | 0.2437 | 0.5508 | 0.3973 | 0.3973 | 0.3973 |
| sparq2 | \| | t1 | 2 | 2 | -0.0461 | 0.0761 | -0.605 | < 0.001 | -0.1953 | 0.1031 | -0.0461 | -0.0461 | -0.0461 |
| sparq2 | \| | t2 | 2 | 2 | 0.7096 | 0.0835 | 8.499 | < 0.001 | 0.5460 | 0.8733 | 0.7096 | 0.7096 | 0.7096 |
| sparq2 | \| | t3 | 2 | 2 | 1.4500 | 0.1137 | 12.757 | < 0.001 | 1.2272 | 1.6728 | 1.4500 | 1.4500 | 1.4500 |
| sparq2 | \| | t4 | 2 | 2 | 1.8895 | 0.1533 | 12.327 | < 0.001 | 1.5891 | 2.1899 | 1.8895 | 1.8895 | 1.8895 |
| sparq3 | \| | t1 | 2 | 2 | -0.1854 | 0.0766 | -2.421 | < 0.001 | -0.3355 | -0.0353 | -0.1854 | -0.1854 | -0.1854 |
| sparq3 | \| | t2 | 2 | 2 | 0.4274 | 0.0787 | 5.431 | < 0.001 | 0.2731 | 0.5816 | 0.4274 | 0.4274 | 0.4274 |
| sparq3 | \| | t3 | 2 | 2 | 1.0491 | 0.0935 | 11.225 | < 0.001 | 0.8659 | 1.2323 | 1.0491 | 1.0491 | 1.0491 |
| sparq3 | \| | t4 | 2 | 2 | 1.7895 | 0.1420 | 12.599 | < 0.001 | 1.5111 | 2.0679 | 1.7895 | 1.7895 | 1.7895 |
| sparq4 | \| | t1 | 2 | 2 | 0.2419 | 0.0769 | 3.146 | < 0.001 | 0.0912 | 0.3927 | 0.2419 | 0.2419 | 0.2419 |
| sparq4 | \| | t2 | 2 | 2 | 0.8871 | 0.0881 | 10.075 | < 0.001 | 0.7146 | 1.0597 | 0.8871 | 0.8871 | 0.8871 |
| sparq4 | \| | t3 | 2 | 2 | 1.5049 | 0.1174 | 12.818 | < 0.001 | 1.2748 | 1.7350 | 1.5049 | 1.5049 | 1.5049 |
| sparq4 | \| | t4 | 2 | 2 | 2.1779 | 0.1963 | 11.094 | < 0.001 | 1.7931 | 2.5627 | 2.1779 | 2.1779 | 2.1779 |
| sparq6 | \| | t1 | 2 | 2 | 0.3090 | 0.0774 | 3.990 | < 0.001 | 0.1572 | 0.4607 | 0.3090 | 0.3090 | 0.3090 |
| sparq6 | \| | t2 | 2 | 2 | 0.9432 | 0.0898 | 10.506 | < 0.001 | 0.7672 | 1.1191 | 0.9432 | 0.9432 | 0.9432 |
| sparq6 | \| | t3 | 2 | 2 | 1.3517 | 0.1076 | 12.559 | < 0.001 | 1.1407 | 1.5627 | 1.3517 | 1.3517 | 1.3517 |
| sparq6 | \| | t4 | 2 | 2 | 1.8372 | 0.1472 | 12.481 | < 0.001 | 1.5487 | 2.1257 | 1.8372 | 1.8372 | 1.8372 |
| sparq1 | ~~ | sparq1 | 2 | 2 | 0.5188 | 0.0000 | NA | NA | 0.5188 | 0.5188 | 0.5188 | 0.5188 | 0.5188 |
| sparq7 | ~~ | sparq7 | 2 | 2 | 0.1775 | 0.0000 | NA | NA | 0.1775 | 0.1775 | 0.1775 | 0.1775 | 0.1775 |
| sparq8 | ~~ | sparq8 | 2 | 2 | 0.1840 | 0.0000 | NA | NA | 0.1840 | 0.1840 | 0.1840 | 0.1840 | 0.1840 |
| sparq9 | ~~ | sparq9 | 2 | 2 | 0.3736 | 0.0000 | NA | NA | 0.3736 | 0.3736 | 0.3736 | 0.3736 | 0.3736 |
| sparq2 | ~~ | sparq2 | 2 | 2 | 0.6456 | 0.0000 | NA | NA | 0.6456 | 0.6456 | 0.6456 | 0.6456 | 0.6456 |
| sparq3 | ~~ | sparq3 | 2 | 2 | 0.3458 | 0.0000 | NA | NA | 0.3458 | 0.3458 | 0.3458 | 0.3458 | 0.3458 |
| sparq4 | ~~ | sparq4 | 2 | 2 | 0.4465 | 0.0000 | NA | NA | 0.4465 | 0.4465 | 0.4465 | 0.4465 | 0.4465 |
| sparq6 | ~~ | sparq6 | 2 | 2 | 0.3635 | 0.0000 | NA | NA | 0.3635 | 0.3635 | 0.3635 | 0.3635 | 0.3635 |
| pos | ~~ | pos | 2 | 2 | 1.0000 | 0.0000 | NA | NA | 1.0000 | 1.0000 | 1.0000 | 1.0000 | 1.0000 |
| neg | ~~ | neg | 2 | 2 | 1.0000 | 0.0000 | NA | NA | 1.0000 | 1.0000 | 1.0000 | 1.0000 | 1.0000 |
| pos | ~~ | neg | 2 | 2 | -0.6230 | 0.0502 | -12.417 | < 0.001 | -0.7213 | -0.5246 | -0.6230 | -0.6230 | -0.6230 |
| sparq1 | ~*~ | sparq1 | 2 | 2 | 1.0000 | 0.0000 | NA | NA | 1.0000 | 1.0000 | 1.0000 | 1.0000 | 1.0000 |
| sparq7 | ~*~ | sparq7 | 2 | 2 | 1.0000 | 0.0000 | NA | NA | 1.0000 | 1.0000 | 1.0000 | 1.0000 | 1.0000 |
| sparq8 | ~*~ | sparq8 | 2 | 2 | 1.0000 | 0.0000 | NA | NA | 1.0000 | 1.0000 | 1.0000 | 1.0000 | 1.0000 |
| sparq9 | ~*~ | sparq9 | 2 | 2 | 1.0000 | 0.0000 | NA | NA | 1.0000 | 1.0000 | 1.0000 | 1.0000 | 1.0000 |
| sparq2 | ~*~ | sparq2 | 2 | 2 | 1.0000 | 0.0000 | NA | NA | 1.0000 | 1.0000 | 1.0000 | 1.0000 | 1.0000 |
| sparq3 | ~*~ | sparq3 | 2 | 2 | 1.0000 | 0.0000 | NA | NA | 1.0000 | 1.0000 | 1.0000 | 1.0000 | 1.0000 |
| sparq4 | ~*~ | sparq4 | 2 | 2 | 1.0000 | 0.0000 | NA | NA | 1.0000 | 1.0000 | 1.0000 | 1.0000 | 1.0000 |
| sparq6 | ~*~ | sparq6 | 2 | 2 | 1.0000 | 0.0000 | NA | NA | 1.0000 | 1.0000 | 1.0000 | 1.0000 | 1.0000 |
| sparq1 | ~1 |  | 2 | 2 | 0.0000 | 0.0000 | NA | NA | 0.0000 | 0.0000 | 0.0000 | 0.0000 | 0.0000 |
| sparq7 | ~1 |  | 2 | 2 | 0.0000 | 0.0000 | NA | NA | 0.0000 | 0.0000 | 0.0000 | 0.0000 | 0.0000 |
| sparq8 | ~1 |  | 2 | 2 | 0.0000 | 0.0000 | NA | NA | 0.0000 | 0.0000 | 0.0000 | 0.0000 | 0.0000 |
| sparq9 | ~1 |  | 2 | 2 | 0.0000 | 0.0000 | NA | NA | 0.0000 | 0.0000 | 0.0000 | 0.0000 | 0.0000 |
| sparq2 | ~1 |  | 2 | 2 | 0.0000 | 0.0000 | NA | NA | 0.0000 | 0.0000 | 0.0000 | 0.0000 | 0.0000 |
| sparq3 | ~1 |  | 2 | 2 | 0.0000 | 0.0000 | NA | NA | 0.0000 | 0.0000 | 0.0000 | 0.0000 | 0.0000 |
| sparq4 | ~1 |  | 2 | 2 | 0.0000 | 0.0000 | NA | NA | 0.0000 | 0.0000 | 0.0000 | 0.0000 | 0.0000 |
| sparq6 | ~1 |  | 2 | 2 | 0.0000 | 0.0000 | NA | NA | 0.0000 | 0.0000 | 0.0000 | 0.0000 | 0.0000 |
| pos | ~1 |  | 2 | 2 | 0.0000 | 0.0000 | NA | NA | 0.0000 | 0.0000 | 0.0000 | 0.0000 | 0.0000 |
| neg | ~1 |  | 2 | 2 | 0.0000 | 0.0000 | NA | NA | 0.0000 | 0.0000 | 0.0000 | 0.0000 | 0.0000 |

**Supplementary Table 2.** Residual Variance.

$Remote

$Remote$cov

rs1_T0 rs7_T0 rs8_T0 rs9_T0 rs2_T0 rs3_T0 rs4_T0 rs6_T0

risq1_T0 0.000

risq7_T0 -0.013 0.000

risq8_T0 -0.039 0.011 0.000

risq9_T0 -0.047 0.011 0.012 0.000

risq2_T0 -0.002 0.143 0.121 0.090 0.000

risq3_T0 -0.125 -0.016 -0.044 -0.029 -0.074 0.000

risq4_T0 -0.069 0.074 0.067 -0.001 0.136 -0.058 0.000

risq6_T0 -0.058 0.030 0.023 0.056 0.132 -0.069 0.027 0.000

$Remote$mean

risq1_T0 risq7_T0 risq8_T0 risq9_T0 risq2_T0 risq3_T0 risq4_T0 risq6_T0

0 0 0 0 0 0 0 0

$Remote$th

risq1_T0|t1 risq1_T0|t2 risq1_T0|t3 risq1_T0|t4 risq7_T0|t1 risq7_T0|t2 risq7_T0|t3 risq7_T0|t4 risq8_T0|t1

0 0 0 0 0 0 0 0 0

risq8_T0|t2 risq8_T0|t3 risq8_T0|t4 risq9_T0|t1 risq9_T0|t2 risq9_T0|t3 risq9_T0|t4 risq2_T0|t1 risq2_T0|t2

0 0 0 0 0 0 0 0 0

risq2_T0|t3 risq2_T0|t4 risq3_T0|t1 risq3_T0|t2 risq3_T0|t3 risq3_T0|t4 risq4_T0|t1 risq4_T0|t2 risq4_T0|t3

0 0 0 0 0 0 0 0 0

risq4_T0|t4 risq6_T0|t1 risq6_T0|t2 risq6_T0|t3 risq6_T0|t4

0 0 0 0 0

$`In person`

$`In person`$cov

rs1_T0 rs7_T0 rs8_T0 rs9_T0 rs2_T0 rs3_T0 rs4_T0 rs6_T0

risq1_T0 0.000

risq7_T0 0.004 0.000

risq8_T0 -0.014 -0.004 0.000

risq9_T0 -0.056 0.030 0.001 0.000

risq2_T0 0.060 0.107 0.006 0.022 0.000

risq3_T0 -0.102 0.017 -0.028 -0.081 -0.055 0.000

risq4_T0 -0.079 0.088 0.050 0.114 0.097 -0.007 0.000

risq6_T0 -0.039 -0.001 -0.066 0.088 0.037 -0.067 0.009 0.000

$`In person`$mean

risq1_T0 risq7_T0 risq8_T0 risq9_T0 risq2_T0 risq3_T0 risq4_T0 risq6_T0

0 0 0 0 0 0 0 0

$`In person`$th

risq1_T0|t1 risq1_T0|t2 risq1_T0|t3 risq1_T0|t4 risq7_T0|t1 risq7_T0|t2 risq7_T0|t3 risq7_T0|t4 risq8_T0|t1

0 0 0 0 0 0 0 0 0

risq8_T0|t2 risq8_T0|t3 risq8_T0|t4 risq9_T0|t1 risq9_T0|t2 risq9_T0|t3 risq9_T0|t4 risq2_T0|t1 risq2_T0|t2

0 0 0 0 0 0 0 0 0

risq2_T0|t3 risq2_T0|t4 risq3_T0|t1 risq3_T0|t2 risq3_T0|t3 risq3_T0|t4 risq4_T0|t1 risq4_T0|t2 risq4_T0|t3

0 0 0 0 0 0 0 0 0

risq4_T0|t4 risq6_T0|t1 risq6_T0|t2 risq6_T0|t3 risq6_T0|t4

0 0 0 0 0

**Supplementary Table 3.** Covariance or correlation matrices among the latent variables.

$Remote

pos neg

pos 1.000

neg -0.676 1.000

$`In person`

pos neg

pos 1.000

neg -0.623 1.000

$Remote

pos neg

pos 1.000

neg -0.676 1.000

$`In person`

pos neg

pos 1.000

neg -0.623 1.000

**Supplementary Figure 1.** Standardized path coefficients

| Remote format |
| --- |
| 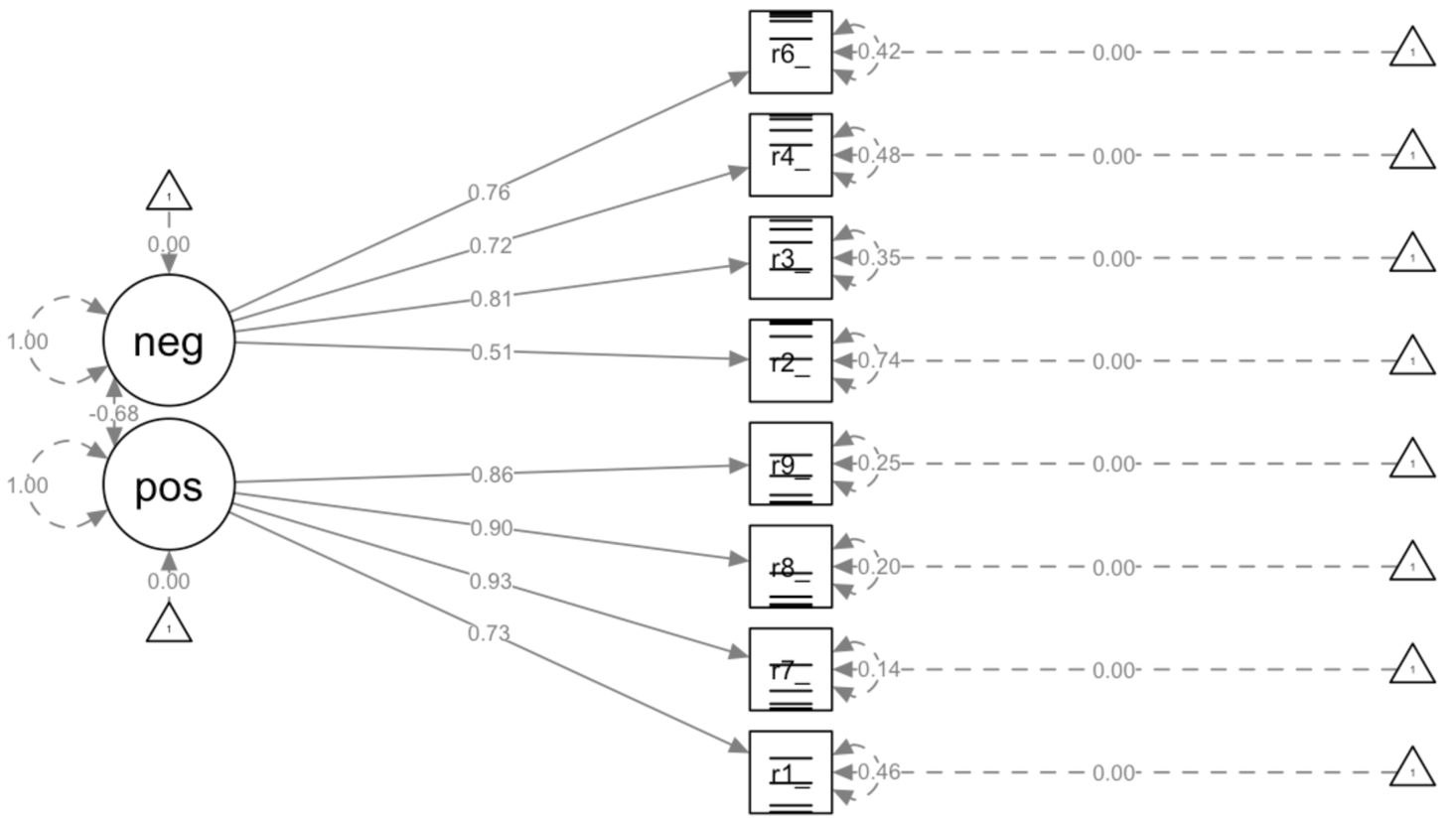 |
| In person format |
| 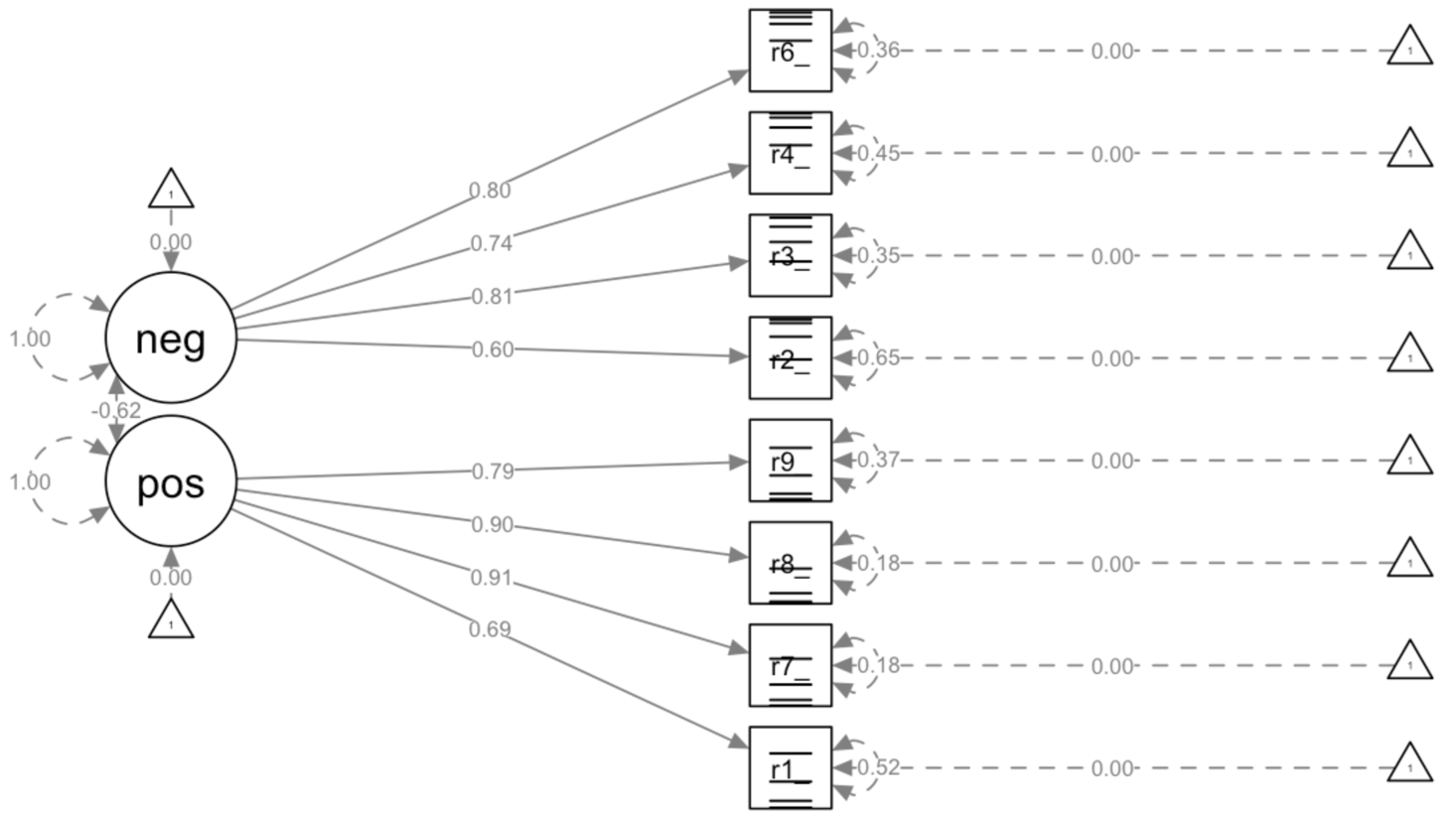 |
